# Reliable Identification Schemes for Asset and Production Tracking in Industry 4.0

**DOI:** 10.3390/s20133709

**Published:** 2020-07-02

**Authors:** Attila Frankó, Gergely Vida, Pal Varga

**Affiliations:** 1 Industrial IoT Division, AITIA International Inc., 48–50, Czetz János u., 1039 Budapest, Hungary; gvida@aitia.ai; 2 Department of Telecommunications and Media Informatics, Budapest University of Technology and Economics, 2, Magyar Tudósok krt., 1117 Budapest, Hungary; pvarga@tmit.bme.hu

**Keywords:** asset tracking, IoT, Industry 4.0, low-cost tracking

## Abstract

Revolutionizing logistics and supply chain management in smart manufacturing is one of the main goals of the Industry 4.0 movement. Emerging technologies such as autonomous vehicles, Cyber-Physical Systems and digital twins enable highly automated and optimized solutions in these fields to achieve full traceability of individual products. Tracking various assets within shop-floors and the warehouse is a focal point of asset management; its aim is to enhance the efficiency of logistical tasks. Global players implement their own solutions based on the state of the art technologies. Small and medium companies, however, are still skeptic toward identification based tracking methods, because of the lack of low-cost and reliable solutions. This paper presents a novel, working, reliable, low-cost, scalable solution for asset tracking, supporting global asset management for Industry4.0. The solution uses high accuracy indoor positioning—based on Ultra-Wideband (UWB) radio technology—combined with RFID-based tracking features. Identifying assets is one of the most challenging parts of this work, so this paper focuses on how different identification approaches can be combined to facilitate an efficient and reliable identification scheme.

## 1. Introduction

The Industry4.0 movement [1] has a huge impact on all manufacturing related fields including logistics and Supply Chain Management (SCM), which are often referred as Logistics 4.0 and SCM 4.0 [2]. While smart manufacturing considered as the main target of CPS (Cyber-Physical System) based digitization trends; logistics—especially resource planning and warehouse management—and SCM can also derive benefits from inter-twining physical and digital planes. A vision of CPS driven warehouses includes autonomous vehicles moving assets from one place to another, based on the data coming from different parts of the supply chain to achieve just-in-time and just-in-sequence delivery. Meanwhile each asset is fully traceable via its Digital Twin counterpart and therefore the inventory updates itself in an automated manner [3,4].

Many different levels of automation exist concurrently in the industry that depend mostly on the size of a company, but the key element has always been the traceability of a product or an asset. This includes not only tracking assets physically within the factory site (e.g., shop-floors and warehouses), but also following their life-cycles and updating, managing their statuses.

The main objective of Productive 4.0, the biggest European research project on Digital Industry to date [5] is to achieve improvement of digitalizing the European industry by electronics and ICT. The three main pillars for the holistic system approach of Productive 4.0 are digital production, supply chain networks and product lifecycle management, all of which interact and influence each other. This current paper reports on an actual use-case of this approach. In this, the results of the digital production (assets) [6] are distributed through the supply chain network (including tasks of warehousing and logistics) [7], and the lifecycle steps [8] of the product can also be monitored through its digital footprint (status and ownership changes get noted at its digital twin). The current paper focuses on the asset tracking aspect of this use-case. The aim of this paper is to cover the gap of the Industry 4.0 movement regarding fully digitalized asset tracking to be deployed by mid-scale manufacturing and logistics sites.

Medium or small-sized companies that usually operate in less, or non-automated environments lack nearly any digital capability to trace products in most of the cases; except administrating the properties of a product on a single sheet of paper attached to the asset. This way of handling products leads to warehouses where finding assets can be a burdensome task because of using *Last In, First Out* (LIFO) stocking method. In such a situation, when it is onerous to locate products or to access them, the preferred solution can be leaving the required asset as it is and searching for—or sometimes producing—a new one which is easily accessible. This approach strongly reduces overall efficiency and productivity, because of increased time of searching for goods and hence increased manufacturing time.

Automated management of asset tracking can make significant improvement in this field by eliminating the aforementioned issues. The key concept is following assets in real-time, which enables us to verify the current location of a piece of product, therefore assets can be fully traceable within the warehouse. This automation can highly enhance the efficiency of logistical tasks, even in environments that do not go for full automation. Most SMEs keep themselves aloof from such a development due to its high cost and the infrastructural changes that may be required.

The current paper presents a cost effective, automated asset tracking and management system which can aid logistical tasks and optimize supply chain management related processes. The solution consist of:

- a real-time indoor positioning subsystem (IPS) based on Ultra-wideband (UWB) radio technology which provides accurate and precise location information;
- an Ultra High Radio Frequency Identification (UHF-RFID) based tracking subsystem using a special identification scheme that is proposed and detailed in this paper;
- the so-called Core system, which handles the information exchange between these two underlying subsystems as well as maintains the asset tracking logic;
- the communication system that allows uninterrupted information flow between the tracked asset’s RFID reader, the IPS system, and the Core system;
- various interfaces to visualization front-ends and external data processing systems, such as ERP (Enterprise Resource Planning) solutions.

Creating a system that is financially worthwhile to adopt for even small-sized companies is one of the main motivations of this work. Therefore, our solution implements an indirect tracking scheme—the IPS subsystem captures the movement of various vehicles and tools (later called as *mule* in short) that can move actual products, meanwhile the tracking subsystem identifies which asset is being moved at the moment. This method is comparably more economical than following each asset individually, but it could also be error-prone due to the difficulties of determining when an asset have been picked up or put down.

The current paper contributes to the state-of-the-art by the following:

- it raises awareness that the Industry 4.0 movement have to keep refocusing to target small-sized and mid-sized manufacturing and logistics scenarios as well, which require the digitalization solutions to have low investment cost;
- it provides an overview of production asset-related reliable identification schemes, and their underlying methods;
- it introduces and details the idea of the indirect tracking scheme, where the assets get associated with the domain where it resides—be it either a warehouse slot or a moving vehicle; and in the latter case the asset’s position indirectly tied with the vehicle position;
- it describes a novel, intrinsically low-cost, highly scalable indoor positioning system—that supports geo-fencing as well as the indirect tracking scheme—which allows the number of trackable assets to be increased by magnitudes without any adjustments of the overall system infrastructure;
- it presents the evaluation results of the system through real-life, practical measurements, especially showing that positioning accuracy can be significantly improved by properly executed simple (low calculation-cost) methods, such as moving averages.

This paper is organized to meet the methodology of information system design science [9] as follows—describing problem relevance and context, related work and gap analysis, system architecture as novel artifact description, evaluation setup, presentation of evaluation results, and finally, the discussion of findings and contribution. Therefore, Section 2 describes the related work including supporting technologies as well as revealing gaps, then Section 3 provides an overview of the system architecture, the concept and the layouts for the measurements, whereas Section 4 details our measurement scenarios used during validation, after which Section 5 evaluates the results related to the identification scheme. Section 6 concludes the paper.

## 2. Related Works

### 2.1. Accurate Indoor Positioning in Real-Time

*Real Time Location Systems* (RTLS)—as its name implies—are used for tracking people and objects at a distance in real-time, while *Indoor Positioning Systems* (IPS) concerns tracking things indoor, where other common localization technologies (e.g., GPS) can not be used. Sometimes the two terms are used interchangeably in indoor positioning related topics, since real-time traceability is a frequent requirement in this field; however, we consistently use the term IPS regarding our system.

Indoor positing solutions rely upon radio frequency technologies in most of the cases; for example, UWB, WiFi, Bluetooth Low Energy (BLE), ZigBee and many others. The chosen localization scheme depends on the application and the used technology, but the most prevalent one—especially in terms of UWB, ZigBee and WiFi—is *ranging* where the distance between two points can be estimated from the propagation properties of radio signals. Ranging includes numerous methods—Time of arrivals (TOA), Time difference of arrivals (TDOA), Angle of arrival (AoA), Received signal strength (RSS) [10,11].

**Figure 1:**
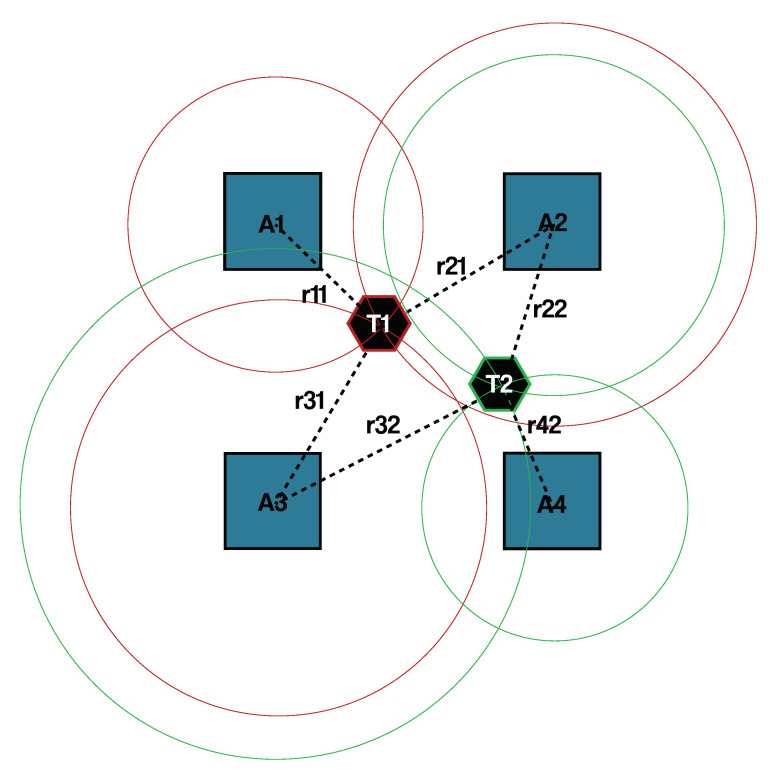
An example system implementing time difference of arrivals (TDOA) and trailateration based localization, where $A_{i}$ -s are anchor points, $T_{i}$ -s are UWB tags and $r_{ij}$ -s are distances.

Our IPS solution uses UWB technology with TDOA ranging method, where distances between nodes can be calculated from time of flight of radio signal. This scheme requires infrastructure that involves reference points, that is, *anchor* points, that have fixed locations. The location of an object to be tracked, that is, *tag* (The term “tag” is used in UWB and RFID nomenclature too. In this paper we will emphasize if it regards to UWB or RFID, each time when it occurs.) can be estimated using trilateration algorithm which use measured distance between an UWB tag and the nearby anchor, as it is shown in Figure 1.

Right now, numerous IPS and RTLS systems exist for various purposes that rely on different technologies as it presented in [12]. In most cases the technology [13] and the localization algorithm determine the main properties of such a system—for example, accuracy, precision, scalability, performance and cost. Since the characteristics of these properties are closely related to each other, only a subset of properties can be optimised within one given implementation. Generally, a better accuracy of a technology or a method implies higher cost and slower response time. In this paper we aim to design and implement a real-time, low-cost asset tracking system which is highly scalable and reliable as well while it is still sufficiently accurate. It is worth to note that there are mixed solution where multiple technologies are used to fulfill different sets of requirements, however these are rather two different implementations that are coupled [14,15].

While Bluetooth, WiFi, ZigBee and RFID based localization techniques are considered low-cost, UWB transceivers are usually more expensive, but this technology can be more accurate than the other ones [16,17,18]. However, the accuracy of a system highly depends on the method itself and other supplementary calculations for example, filtering, predictions [19,20,21] and auxiliary units (e.g., IMU-s, cameras) and supporting technologies [22,23]. There are plenty of methods to improve the accuracy of the aforementioned techniques, but generally speaking these methods often require much more computational capacity or more complex and dense infrastructure. Contrarily, a TDOA method based on UWB can provide an accuracy of 30 cm without any further calculations, while the response time of the system is still really fast—for example, even under 100 ms, considering a cheap (under $5) controller unit. Moreover, the complexity and the density of the infrastructure remains low, compared to other solutions. It is also well known that UWB has much larger power consumption than Bluetooth or ZigBee, however its energy efficiency is better, therefore it is more sufficient for high-data rate applications—for example, providing real-time, highly granulated location information as an Industry 4.0 compliant solution [24].

### 2.2. Radio Frequency Identification in Logistics

Radio-Frequency Identification uses electromagnetic waves to track or identify certain objects that RFID tags are attached to. The data stored on *passive tags* can be captured and read by an active RFID reader module which interrogates and supplies tags by transmitted electromagnetic waves. Tags do not need to be within direct line-of-sight of the reader so it may be embedded in the tracked object [25]. On the other side *active tags* transmit electromagnetic waves, so they have much longer range and can be interrogated in a more reliable way, although they are supplied by a battery and have a significantly higher price tag.

The range of a High-frequency (HF) RFID reader is usually between 10 cm and 1 m which is acceptable for identifying for smart cards, but it is inappropriate in use-cases where the distance between a reader and the assets can not be decreased due to physical boundaries. In contrast to this, the range of UHF-RFID reader is between 1 m and 10 m—that can be expanded to 100 m by using active tags. Moreover, UHF-RFID standard has an extra capability that is not supported in HF-RFID, the *simultaneous* or *bulk reading*. During bulk reading reader is able to interrogate multitude of passive tags simultaneously in one reading cycle which can be extremely useful in logistical use-cases where the individual assets are often bundled together. The hit-rate of the method—the ratio of identified (read) tags to all tags—depends on the performance of the reader and the positions of antennas as Ustundag et al., presented in Reference [26], therefore it seems the higher the required hit-rate, the higher the cost of the reader. However, hit-rate can be improved with different methods such as adaptive time and power control too [27].

It is worth to note that RFID itself can used as core technology for an IPS system along with different methods. The most trivial one is based on active RFID tags [28], but there are numerous solutions based on passive ones. However, these systems do not provide the desired accuracy or require dense infrastructure [29].

Radio Frequency Identification is often considered as one of the core technologies of Internet of Things paradigm, especially regarding industrial usage. It is commonly used in logistical task such as identifying products, tracking items, tools and pieces of equipment (not location, but their presence). Most of these applications aimed to facilitate RFID based management systems that synchronize with company’s ERP system to keep the inventory up-to-date [30,31,32]. These systems can highly improve the efficiency of logistics by eliminating manual updates; however, their scope is limited and they can be used only in environments where only few special locations (e.g., workstations, gates) are equipped with identifying capabilities, but not in dynamically changing ones.

### 2.3. Asset Tracking Approaches in IIoT

A decade ago, localization and asset tracking was almost synonymous, since the set of assets—the collection of objects to be tracked—was static, and they were tracked directly. Nowadays, asset tracking means more than localization, since it also includes identification of the assets to achieve tracking in an environment where the set of assets is dynamic and always changing. Therefore in most of the current asset tracking approaches, it is required to couple localization and identification technologies to work together, but in certain cases one technology can serve both purposes—usually at the expense of precision and accuracy. However, in this paper we propose an UWB and RFID based asset tracking solution, it has to be emphasized that different use cases require different asset tracking approaches. There are numerous factors that affect the properties of an asset tracking system such as: the size of the area of logistical operations, the infrastructure within this area, the number of assets to be tracked, the required accuracy of location, the maximum affordable cost of the system, how easily can the system be deployed, and many others.

Most asset tracking systems share the same kind of infrastructure where reference points must be deployed within the tracked area while the tracked assets are equipped with tags [12]. In the case of such an infrastructure the price of a Bluetooth based system is always lower (approx. half a price) than an UWB [33] or WiFi based system [34], since the price of a BLE beacon varies between $10 and $20 [35,36,37]. Moreover, these type of beacons do not need any auxiliary components to operate, while UWB transceivers require a microcontroller unit that commands them. Since they are often bundled onto a PCB, there are additional manufacturing costs when compared to the BLE based solution.

Most BLE or purely RFID based solutions aim for power-efficiency or simplicity when compared to other ones that use coupled technologies. Although these simpler and more economical systems only provide an accuracy of 1–2 m, this is adequate for various logistics-related or other uses cases [38,39], especially where humans are involved in the scenario. These systems are often aided with different algorithms such as Kalman-Filter to provide a better accuracy [40], however in this case their response time becomes higher, therefore real-time systems can not be implemented this way. Some low-powered solutions apply custom approaches to achieve energy-efficiency and to keep the costs down low, but their characteristics are very similar to BLE based systems—mostly regarding to accuracy and precision as well as the required density of readers [41,42].

UWB based solutions are usually coupled with RFID or UHF-RFID technology, since the first one provides high accuracy with a short response time and the second one is a relatively cheap technology that can enable identification or large number of assets—while some applications use both of them to localization [14,43]. Most of these asset tracking solutions based on that approach, where tags are capable of communicateing via UWB as well as RFID [44,45,46,47], so one device can serve both purposes. While these systems are considered the state-of-the-art for on-site deployments, their scalability and generic applicability is limited when it comes to economical considerations.

The main disadvantage of the previously described only-UWB and hybrid UWB/RFID approaches is that each asset has to be equipped a tag, so a large number of assets can highly increase the cost of a solution, while other solutions that rely upon only RFID technologies are more scalable in the manner of cost, since the price of an RFID tag is 10 cent or less. In this case static readers can identify assets and determine their locations, although it requires plenty of readers which prices can be really expensive depending on the used frequency (HF or UHF) [48].

Hence, a research gap for a scalable, economical asset and production tracking solution needs to be covered for wider applicability of Industry 4.0 results in small-sized and mid-sized stakeholders of the value chain.

There are numerous RTLS and tracking system on the market right now with different capabilities and costs to serve different purposes. In this paper we introduce a different tracking approach which unifies the advantages of the aforementioned approaches. In this case the assets are tracked indirectly, therefore the required infrastructure do not have to scale with the number assets. This means that the cost of the system could be as low as possible to make it an Industry 4.0 compliant asset tracking solution that can be applied at minor companies too as a tool of digitization across the value chain.

### 2.4. Interoperability and Sharing Information within System-of-Systems

There are many players of the ecosystem in which the smart asset physically (or virtually) gets moved between manufacturers, integrators, transporters, distributors, owners, and other stakeholders. Data exchange among these should be fluent, at least when it comes to information sharing of the smart asset status (regarding location, ownership, environmental parameters, etc.). The systems that are involved in keeping and updating the status at a given stakeholder—or passing to another one—should be able to share information; but it traditionally have various obstacles, including compatibility of communication capabilities and security policies. The Arrowhead framework has been created for addressing exactly these issues, and in respect to integrability and interoperability, the Arrowhead framework really sticks out from other Industrial IoT initiatives [49].

In order to enable flexible system connections in a dynamical manner, Arrowhead enforces the application of Service Oriented Architecture (SOA) concepts. In these, information exchange between systems are established through services—so systems can provide and consume services. The main features of SOA include late binding, discovery, and loose coupling, which means that communicating entities are not connected to each other design or configuration time, but they discover each others servicing capabilities run-time, and then remain only loosely coupled: they (can) disconnect from each other after the information exchange and sing another peer for the next servicing step [50].

**Figure 2:**
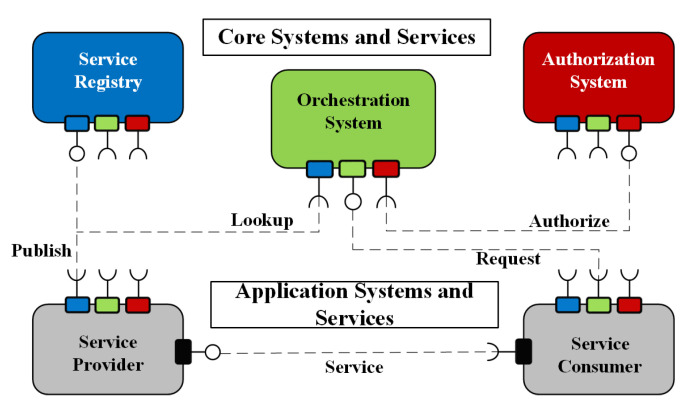
The core elements of the Arrowhead Framework are connected in a service-oriented way.

**Figure 3:**
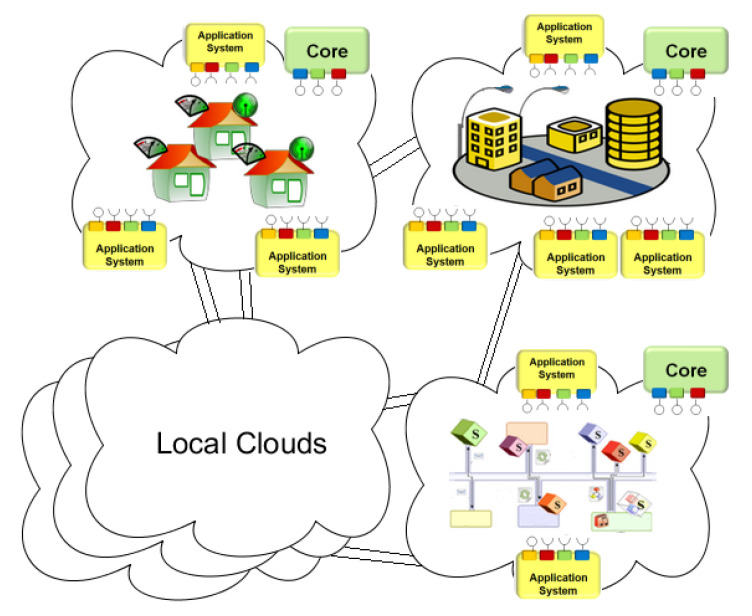
Local clouds with serving different industrial domains have their internal requirements (such as latency or security) guaranteed—and they can produce and consume services to/from other Local clouds as well. [51]

Another feature of the Arrowhead framework is the concept of Local Clouds [51]. The main idea here is that industrial systems have special requirements—in latency, security, engineering complexity, and so forth—when compared to general IoT. These requirements make the "global" IoT platforms and clouds impractical, and requirements are hard—or impossible—to meet for the industrial players. Hence local clouds can be defined for the System-of-Systems that communicate on the manufacturing floor, others on the warehouse, yet another ones on the enterprise planning level, or in the logistics network, and so forth. Service producers and consumers are orchestrated together within the local clouds with the help of mandatory core systems defined by the Arrowhead framework—such as Service Registry, Orchestrator, or the Authorizer system, as shown by Figure 2. Furthermore, the inter-cloud servicing is introduced as a novelty, and also supported [52] practically as well. Inter-cloud servicing is a necessary concept for flexible but secure information exchange between the systems of different stakeholders—so service consumers and producers can connect even if they reside in different local clouds, as Figure 3 shows.

**Figure 4:**
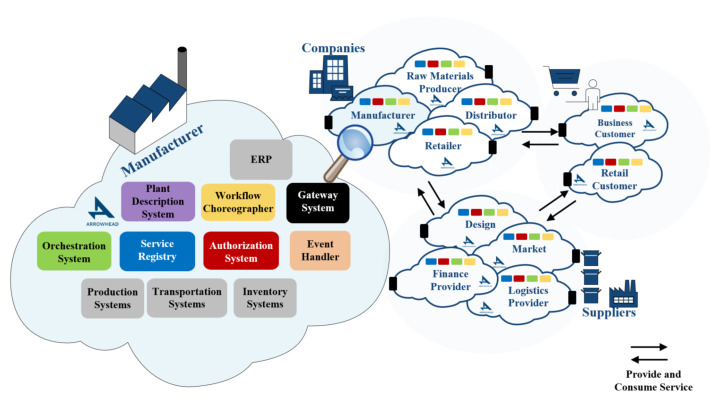
System-of-Systems established throughout the supply chain network can exchange information in a service oriented architecture. [7]

The high-level interoperability concept for the entire supply chain—that is supported by Arrowhead—is visualized by Figure 4 [7].

## 3. Overall System Architecture

### 3.1. Localization Scheme and General Workflow

The infrastructural and architectural design of our system is based on well-defined requirements which are derived from certain use-cases as it was described in Reference [53]. The system itself uses UWB technology and implements a TDOA based localization scheme. Both anchors and UWB tags are pieces of a unique hardware; however, only UWB tags are capable of reading passive UHF-RFID tags.

**Figure 5:**
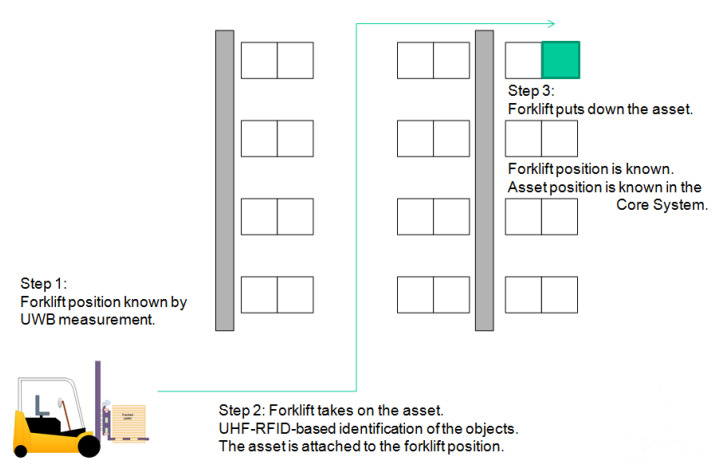
An example scenario for asset tracking with integrated UWB-based indoor-positioning and Ultra High Radio Frequency Identification (UHF-RFID)-based asset identification.

An example scenario for the overall motivation is shown by Figure 5. In this use-case a forklift moves inside a warehouse and its position (determined by the IPS with the help of UWB) is getting associated with assets that it picks up (attach through UHF-RFID reading) or puts down (detach through loss of RFID signal).

Since anchors are the reference points, their location is fixed: they are usually deployed on walls or surfaces of static objects, meanwhile UWB tags represent the vehicles or tools that can replace assets to be followed which we can call mules in general. In this case the only restriction regarding to the placement of the devices within the tracked object is: it must not be placed in shielded area (e.g., under metal surfaces).

The workflow of the system relies on the tight coupling of localization and identification. The localization method follows the general scheme where the relative position of an UWB tag can be calculated from the measured distances between the UWB tag and the nearby anchor points. This relative position can be converted into an absolute position if the locations of anchors is known in the tracked area. The calculation and conversion are processed by the so-called *Core System*, which also handles the trigger events and command messages.

The identification scheme is basically trigger-based and event-driven; in other words: the system identifies an asset only if some specific conditions are met. Events are changes in the physical world such as when a mule starts or stops of its motion. These events can be detected by various sensors that generates trigger signals to command the RFID reader on the mule to read nearby RFID tags. The set of events that can be used as triggers is limited and highly depends on the use-case, but generally picking assets up and putting them down are the core events that can be possibly found in any circumstances.

Full traceability of assets is provided by the unified localization and identification workflow. The IPS subsystem tracks each mule and object that can relocate products or assets. When an asset has been picked up, it triggers the identification subsystem which identifies the product or products and commands the Core system to attach the read RFID tags virtually to the tracked mules. Similarly, when an asset has been removed from a mule, the identification subsystem commands the Core system to detach the related RFID tags from the tracked object. This scheme is an indirect form of asset tracking, since we do not follow the asset itself, but the mules that can move them.

Within this scheme, movement of assets can be traced even if they are not tracked directly; however, the corresponding vehicles and tools are. We call this scheme *indirect asset tracking*, since we do not follow the asset itself, only the mules that can move them. This approach enables our solution to be inexpensive and easy to deploy by avoiding the usage of more complex methods and infrastructure regarding asset identification, because the number of assets does not really have an impact on the size of the infrastructure. Moreover, due to this indirect scheme the system is scalable in a wide range, and expandable as well, therefore it fits well into medium-, and small-sized companies’ workflow. An example for this workflow applied in a warehouse scenario is shown by Figure 5.

### 3.2. Identification Techniques and Methods

Since asset identification is a fundamental part of the system and it determines its overall efficiency, we have to ensure that it is reliable and fault-tolerant. The used scheme assumes that only those relevant RFID tags are identified which have been actually put onto the mule or removed from it. Here, multiple approaches can be applied to set different conditions and rules up which can serve as basis to distinguish between valid—to be followed—RFID tags and static nearby tags.

Event-driven identification is the main approach that we used and will be detailed in further sections. Another approach can be the *continuous reading*, when the RFID reader continuous polls nearby tags without any trigger. In most of the use-cases the unified UWB tags and RFID readers (hereinafter referred to as *AT devices*) are installed onto vehicles (e.g., forklifts) or other objects that can replace assets. In such an environment the AT devices can be supplied only from external battery source or the vehicle itself which involves reducing power consumption as a critical factor. According to this requirement, *continuous reading* methods can not be used because of their high energy demand.

For example, according to Reference [54], the average power consumption of an UHF-RFID reader is 3.25 Watts assuming a supply lane of 5 VDC. In the case of supplying from a battery that has an appropriate size (smaller or equal to the AT device itself)—which means that its capacity is a maximum of 5 Ah and its peak voltage is 14 V (assuming commercial LiPo battery)—the estimated number of working hours is about 17–19 hours, which is not acceptable. In the case of supplying from the vehicle, the AT device highly reduce the working hours of vehicles that leads to more frequent charging periods, which is also not desirable from logistical perspective. Note: Peak voltage reduction of battery from 14 V to 10 V is included.

Trigger-based identification can be implemented in many different ways—this paper focuses on geo-fencing and motion based triggers. The term geo-fencing covers defining virtual perimeters for real-word geographic areas to enable the usage of location-based services. Using geo-fencing is beneficial in those environments where boundaries of staging, storing, loading or other special areas are well-defined [55]. In this case, these areas can be fenced virtually and if an AT device enters or leaves a pre-defined, geo-fenced area, the RFID reader will be triggered to scan which assets entered or left the area [56]. This method can be useful when geo-fenced areas cover the full map, although it provides less accurate asset location data.

Motion-based methods refer to schemes when events related to the movement of AT devices (e.g., start and stop) trigger identification. The main difference between these schemes is the source of trigger that is, how these events are detected. One approach is the position based detection scheme, where the source of trigger is our existing infrastructure—the movement of AT devices can be estimated based on the real-time location data-streams. The other approach includes the usage of external trigger sources such as separate modules or attached sensors—for example, Inertial Measurement Unit (IMU) [57]. These units provide information about an object’s special force (acceleration) and angular rate, so it can be applied as a supplementary unit for tracking [58], but this technique is based on sensor fusion which is computation heavy process. Therefore, in this paper we use a different approach, since most of these units can also detect and sign specific event like start and stop of movement. These sensors are really sensitive to even minor movements hence they detect motions that should not be tracked (e.g., small movements during loading), so we have to eliminate these false-detections in our identification scheme.

### 3.3. Unified Identification Scheme

**Figure 6:**
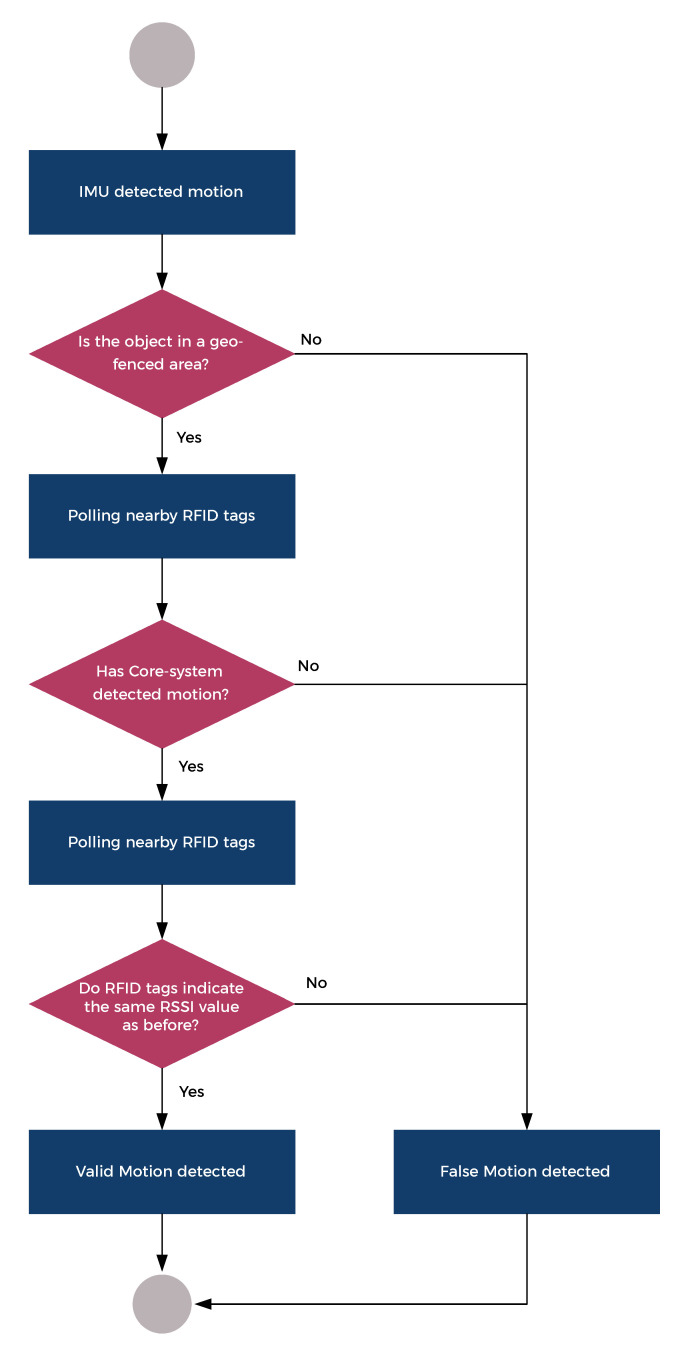
Detection tree of a START event.

Each identification scheme has different advantages and disadvantages, so they do their best in different applications. While geo-fencing is advantageous when there are well-defined logistical areas, it can not be used if an asset can be relocated to anywhere in the warehouse. In contrast to this, motion-based schemes fit very well into such an environment, but they err more, since start and stop events occur when a mule stops with load for any other reason than putting it on or removing it. Triggers that are generated by an event which is different than loading and unloading are called false detection in this scheme. To get the most out of this system, a combined scheme has to be used which unifies geo-fencing and motion-based schemes to create triggers for the RFID reader as can be seen in Figure 6.

The following elements are the part of the combined scheme:

- **Geo-fencing** based trigger generation: It can be detected if an AT device enters or leaves an area.
  - Advantages: Out-of-the-box method (no further costs), ideally no false detection;
  - Disadvantages: Works only in well-defined environment, asset location accuracy depends on the size of the areas, ranging error can cause false detection.
- **Position** based trigger generation: Based on the real-time location data, it can be determined if an AT devices is moving or not.
  - Advantages: Out-of-the-box method (no further costs), provides really accurate asset location;
  - Disadvantages: false detection due to start/stop events while mule is loaded, false detection due to ranging error.
- **External source** based trigger generation: Based on the external sensor such as IMU-s, it can be determined if an AT devices is moving or not.
  - Advantages: provides really accurate asset location, independent of ranging error;
  - Disadvantages: false detection due to high sensitivity and start/stop events while mule is loaded.

It is worth noting that non-event driven approaches—for example, **periodic polling**—can be used as supplementary methods. Periodic polling is also a great way to eliminate false detection when geo-fencing is not an option, because assets can be unloaded anywhere in the warehouse. In this case the Received Signal Strength Indication (RSSI) values are used to determine the distance of certain RFID tags from the reader. This can be applied as a filter during movement to avoid false detection: if distances of RFID tags are the same before stop and after start, then RFID tags should not be detached from the AT device.

## 4. Measurements

### 4.1. Overview

To achieve the full potential of our unified identification scheme, we have to refine the position based method, in particular, what type and amount of motion is considered as moving. If an AT device moves 50 cm back and forth during loading, for instance, it does not count as moving.

A major issue here is false detection due to ranging error—this occurs if the absolute distance error—the distance between the physical position and the estimated one—is greater than the value that we defined as the act of moving previously.

**Figure 7:**
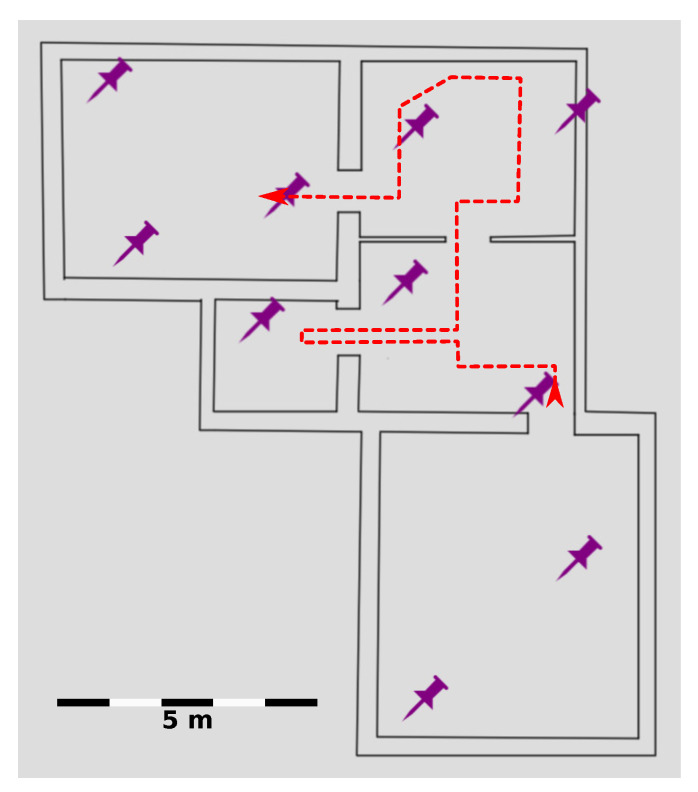
A schematic map of the test environment for measurements, where purple thumbtacks are anchors.

This approach requires to measure the limits and characteristics of the presented solution to see if the application is capable of using the unified method without any modification. Each measurement was performed indoors with a static layout that is shown in Figure 7.

### 4.2. Overall Accuracy of Ranging

**Figure 8:**
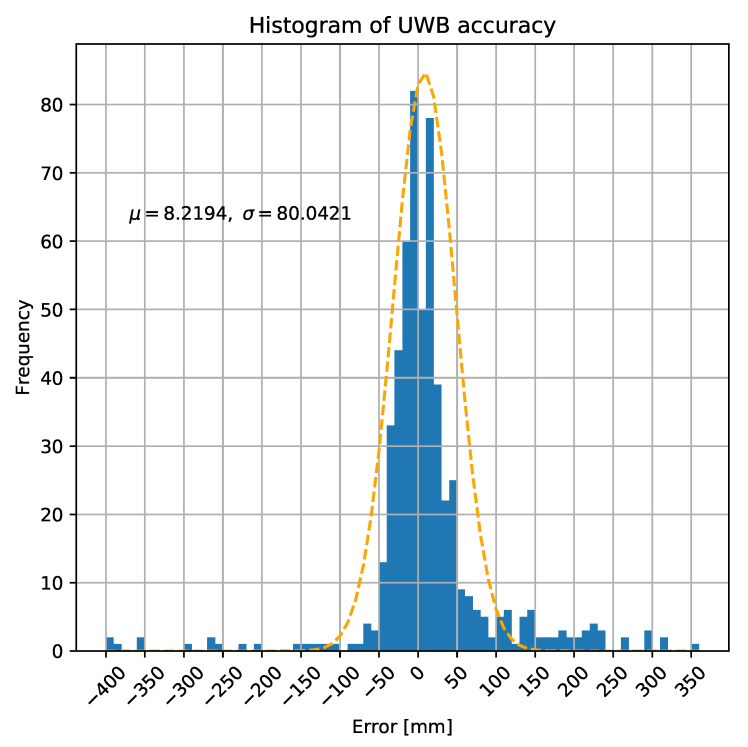
Statistical characteristics of UWB ranging.

First, we measured the overall accuracy and characteristics of our UWB ranging implementation as shown by Figure 8. In this scenario merely the distance was measured between two distinct, static UWB devices. According to results—which contains 566 samples—the excepted value of the error is 8.22 mm and the standard deviation is 80.04 mm. This implies a really good overall accuracy—in 482 of 566 cases (85.1%) the value of error is below 80 mm (one sigma)—and 98.5% of the sample has an error which is less than 300 mm, so the average amount of error and the dimensions of tracked vehicles and tools (e.g., forklifts or pallet trucks) differ by more than one order of magnitude. However, these results enable the usage of the position based method (since relatively tiny movements can be tracked), we have to deal with the outlier values later on.

### 4.3. Start/Stop Events—Definition and Detection

Defining start and stop events is not a straightforward task, since these definitions are highly depend on the actual size of a vehicle or a tool. For example: a common forklift is 3.1 m long and 1.5 m wide, whereas a pallet truck is 1.5 m long and 0.5 m wide, therefore a displacement of 2 meters may trigger a start event in the case of a pallet truck, but not for a forklift.

To determine a minimum of displacement that can be used as a trigger in this application, the overall error—that includes ranging error and inaccuracy of triangulation—has to be measured within another scenario. In this case, there are multiple number of anchors and one UWB tag is to be localized.

**Figure 9:**
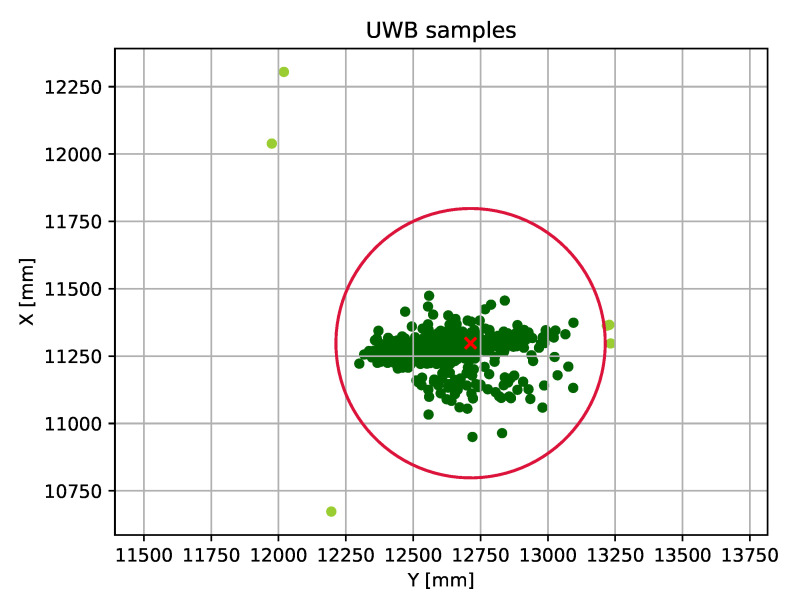
Position dataset created by a static device.

As it is seen in Figure 9, the calculated location dataset has much worse accuracy than the previous one that contains only raw distances. In this case the expected value of error is 167.85 mm, while the standard deviation is 95.78 mm, based on 1449 samples.

This means that the minimum of displacement that the system can use as a trigger is approximately 0.5 m (red circle in the figure), derived from the measured results. As it was described, most of our objects to track have significantly bigger dimensions than the average value of error, therefore this scheme is still applicable and reliable (with having in mind that we have outlier values), however, the overall efficiency can be increased by refactoring the trilateration algorithm.

### 4.4. Elimination of the Outliers

The above-mentioned scheme still suffers from a major issue that can reduce the feasibility of the whole system, the outliers. As it is shown in Figure 9, most of the position data can be found within a circle with a given range, but there are also several values that include a higher offset due to the error.

**Figure 10:**
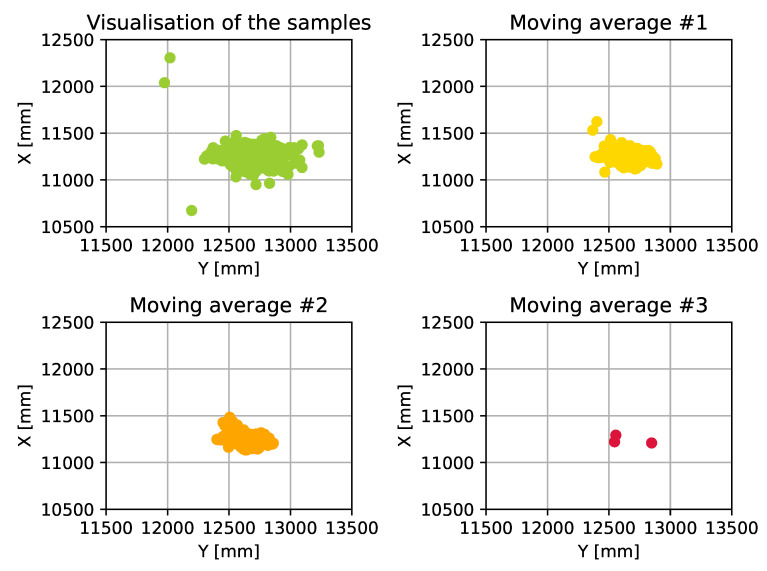
Using moving average with different window sizes.

Elimination of these outliers requires the filtering of the calculated datastream: this can be executed during the trilateration process (e.g., by using Kalman-Filter [59]) or after the calculations. In these measurements we used moving average with three different window sizes to reduce the amount of error and eliminate outliers. The first option (#1) uses the last three value for the calculation, the second option (#2) uses a wider window, where the previous two and the next two values are used, while the third option (#3) uses the same, but only displays new values when the difference is bigger than 30 cm. Figure 10 visualizes our results.

**Figure 11:**
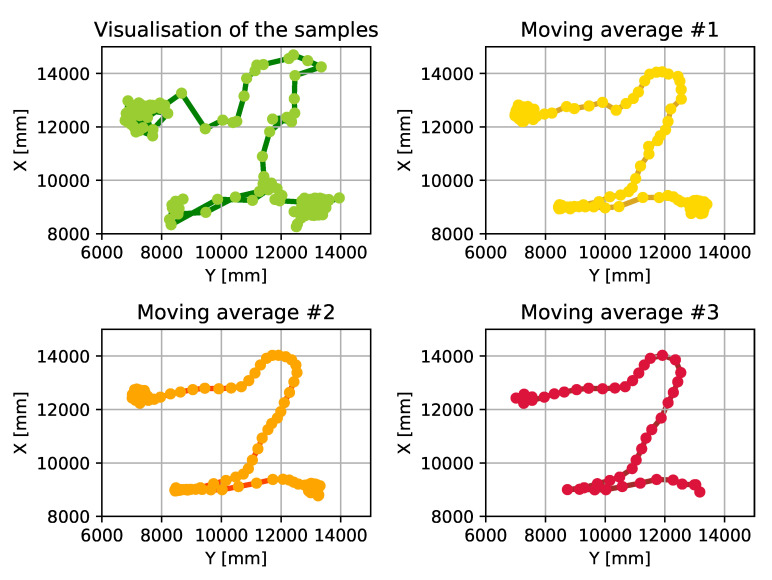
Using moving average with different window sizes on a path tracked by a moving device.

As can be seen in Figure 10, the usage of moving average eliminates the outliers to keep the values of the dataset within the defined circle. It is also shown in the figure that how the different moving averages affect the dataset and reduce the size of the covered area. While it seems like choosing a quite large window size for the moving average can be the best choice for the system to meet the requirements, we have to consider that the AT devices are moving object. Therefore it is mandatory to run this algorithm on a dataset where the device is moving. Figure 11 shows the result of the algorithm when it is used in a scenario where the AT devices is in motion. As this figure shows, there can not be found any significant differences between the used window sizes: each method smooths the displayed route, but none of them cut important parts out in order to achieve full traceability—within the defined requirements.

## 5. Evaluation of the Identification Scheme

As we discussed in previous chapters, our main goal is to achieve full tracability of assets. This traceability enables the system to be applied within various logistics tasks across the whole supply chain as an automated asset tracking solution. To ensure that our system meets the requirements, the indirect identification scheme—which is one of the core functionalities of the system—has to be tested and validated.

### 5.1. Measurement Setup and Test Scenario

The following measurements were executed in the previously described environment. The main purpose of these tests is to evaluate the identification scheme—we simulated a basic scenario, where a forklift carries some of the goods between two workstations in a factory. The forklift is equipped with three components of our system:

- UWB based positioning subsystem: the forklift is continuously being positioned by its UWB tracker,
- RFID reader: the forklift has an RFID reader on its front to identify the carried goods,
- IMU: the accelerometer detects the current state of motion.

The test scenario consists of three phases which covers all the possible cases that are handled by the identification scheme, while being executed within a short time interval and physical path. In the first phase the forklift arrives at *workstation A*. At this station there are goods that should get loaded onto the mule and transferred to *workstation B*. After stopping at the station A, in the second phase the forklift follows its schedule and takes the goods to workstation B. At the halfway point of the path, the forklift stops as it faces temporary obstacles. When the path is free again, the forklift continues its motion to workstation B. After arrival, in the third phase the transferred goods get unloaded and the forklift leaves the station. To simulate a realistic scenario there are additional goods both on workstation A and B. These boxes can be also tracked by the RFID reader of the forklift.

### 5.2. Visualization of the Test Results

#### 5.2.1. Positioning and Motion Detection

**Figure 12:**
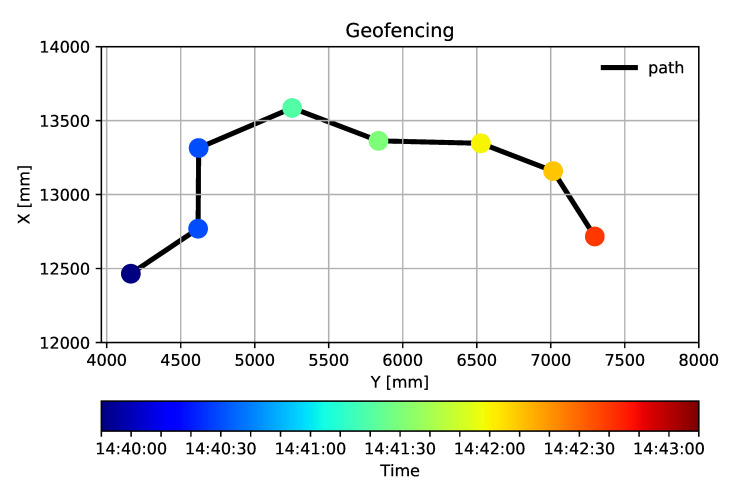
The path of the forklift.

The measurements take place in a small warehouse, while the processed part of the data series were collected during a 3-minute interval. Figure 12 shows the path of the mule based on UWB positioning. The presented samples are colored according to the time scale below the graph. The measured position data is filtered by the previously described position-based technique. Firstly, it is smoothed by moving averaging to eliminate outliers. After that it is filtered to separate the significant position alteration from the local movements: every presented position is farther than 50 cm from the previously calculated one.

**Figure 13:**
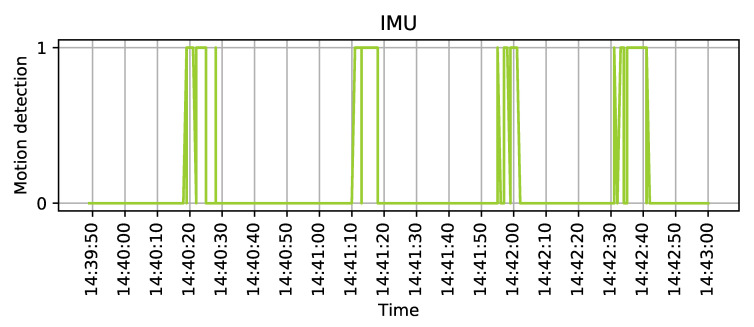
Inertial Measurement Unit (IMU) triggers the system by setting "1" its output.

The three phases of the test are also identifiable in the figure—the first phase is marked with three shades of dark blue, the second phase is separated in light blue (before the obstacle) and yellow (after continuing the schedule). The third phase is presented in darker orange. The timetable of the movements is summarized by Table 1:

The three phases are also visible in Figure 13—the graph shows the data collected by the accelerometer. The four significant peaks mark each listed event from the table. In the demo setup, IMU subsystem is providing a simple one-bit trigger (interrupt) for the system, to reduce the computational cost to the minimum possible level. On the contrary, UWB ranging is a more complex operation that uses more resources to provide a more sophisticated output.

#### 5.2.2. Asset Tracking

**Figure 14:**
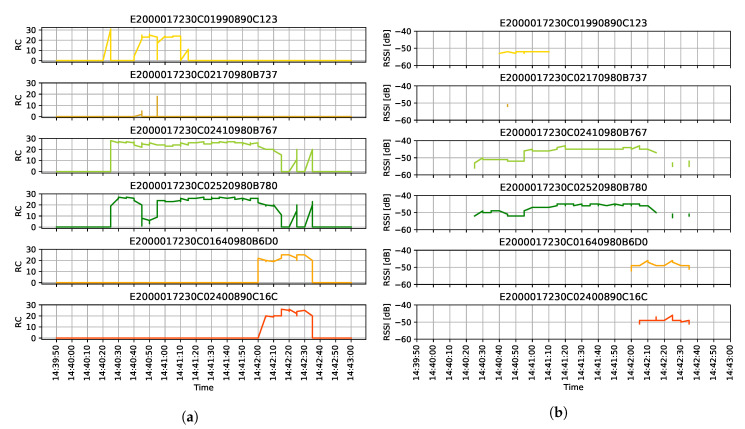
RFID measurements. (**a**) The Read Count of the tracked RFID tags. (**b**) The RSSI value of the detected RFID tags.

Our system identifies the transferred goods by tracking the RFID matrices on their cover. In this test scenario, 6 different tags were used: 4 on boxes at workstation A and 2 on workstation B. The task of the forklift is to transfer two of the boxes from A to B while the RFID reader permanently updates its information on the currently trackable tags. This process is clearly traceable in Figure 14a,b: the tracking of matrices not only identifies the boxes, but also provides information for the identification scheme by measuring RSSI and Read Count.

As it was shown in the position data, the RSSI and Read Count also follow the three phases of the test scenario. The RFID reader starts tracking the matrices when the forklift arrives at workstation A and two of them are continuously being identified in the next two minutes. When the forklift is reversing from workstation A, it loses the signal of the remaining two. As the forklift is heading towards workstation B, the reader identifies 2 more RFID matrices, and keeps on tracking until it leaves the station.

### 5.3. Data Analysis and Outcomes

At the start of the demo, the system operates in stand-by mode, until the IMU detects a change in the state of motion and fire a trigger. If an AT devices is in sleep mode, the trigger wakes the AT device up. Firstly, the UWB tag validates the trigger of the IMU by providing its position to the core system: the new position has to significantly differ from the previously calculated one, otherwise the system sets back to stand-by mode. This step is fundamental to ensure that the difference is caused by a real motion and not only some error of the positioning system. It is also worth to note that, stand-by mode does not necessarily means that the AT device is sleeping, but it is not in motion. After validating the trigger, the RFID reader periodically polls the RFID tags within its range to identify the transferred goods. When the forklift stops, the UWB positioning system indicates the steady-state of the position and sets the stand-by mode until the next trigger. This sequence of system operations is identifiable in Table 2 where a summary of the collected data is presented. The first column of the table lists up the time intervals which is followed by the columns of IMU triggers and UWB positioning. The fourth column records the number of tracked assets and the last two columns contain the current mode and asset tracking events based on the collected data.

In Table 2 there are five identifiable phases in contrary to the previously described three phases of the test scenario. This difference roots in the different approaches of positioning and asset tracking. IMU and UWB data are following the changes in the state of motion. From this point of view, the process consists of five phases, separated by the IMU triggers. However, some of these information is irrelevant regarding asset tracking. For example, when the forklift temporarily stops at the obstacle, the load is unchanged. This is clearly traceable in the RFID data: the reader tracks 2 tags constantly between A and B (*E20...767* and *E20...780* in Figure 14a,b. In general terms, asset tracking events merge the five phases: the last column of Table 2 indicates that valid events happened at the two workstations, while the stop at the obstacle is irrelevant.

As it is shown, the tracking scheme is reliable since the assets that are relocated are fully tracked between the two workstations despite of the several intermediate stop and start events. According to this, the system can serve its purposes as a low-cost tracking solution for certain logistic tasks across the whole supply chain.

## 6. Conclusions and Future Work

Deployment of automated inventory management systems and asset tracking solutions is an important step on the way of enhancing logistics in order to reach full digitization of this field. Nevertheless, production and asset tracking can highly improve overall efficiency and productive even in less automated environments.

This paper described a novel method—and a corresponding reference system implementation—that utilizes technologies UWB and RFID in an integrated way to implement an automated management solution for asset-tracking. The method uses an indirect tracking scheme where the vehicle or object that can relocate assets are followed instead of the assets itself. Using the UWB technology ensures that the system is accurate enough for warehouses of small and medium sized companies without significant computation requirements, so the location information can be provided in real-time. Since the assets are equipped only with passive RFID tags which do not affect the size of the infrastructure, the cost of the system remains low, while it still highly scalable due to the UHF-RFID technology.

The most significant disadvantage of the aforementioned method is that the asset tracking scheme can be error-prone because of the indirect approach. In order to make the implemented systems reliable, our method uses an identification scheme based on different trigger sources and filtering methods to eliminate false detection and to provide accurate location information of any asset, in a reliable way.

As it has been already shown, this indirect identification scheme is highly reliant upon the mules that can relocate various assets. Therefore the usage of the presented system is limited to such arrangements where merely a set of vehicles or tools can actually move the objects to be tracked, since each of the mules has to be equipped with tag devices. Other limitations are derived from the underlying technology stack—the current UHF-RFID readers can handle only approx. 200 RFID tags simultaneously [54], which maximizes the number of assets that can be relocated together as a batch. This is a theoretical maximum, however, it highly depends on the actual reader—which may provide higher rates then the aforementioned one [60]. Additionally, while we do not have to deal with density issues related to UWB, but the usual limitations of such a wireless technology—shielding metal surfaces, certain amount of interference—still affect the system. This, however is a common challenge for all indoor-localization systems. Together with the description of the method and the utilized technologies and integrated systems, the paper presented a concrete proof of concept scenario as well, to demonstrate how the indirect asset tracking scheme works and how reliable this concept really is in a specific use case.

To summarize the evaluation results, the system is an efficient implementation of asset tracking due to the cooperation of the different subsystems and technologies and the presented identification scheme. The main advantages of the system are its high scalability, and the possibility of continuous adaptation. When applying our indirect identification scheme, the number of assets to be tracked is not bound to the overall infrastructure of the system, thus the number of assets can be increased by magnitudes without extending the infrastructure itself. Moreover, the architecture itself is modular, so if there is a need for extending the number of tags (mules) or anchors, it does not require significant financial effort, since each new device can be added to the system one by one. Similarly to other state-of-the-art systems, this one supports energy-efficiency as well, by involving inactive periods during stand-by mode that lower power consumption of the RFID reader, while there is no information loss, because all of the necessary asset identifications happens in motion (unnecessary measurements are in brackets in Table 2). Furthermore, forklifts operate as searchlights in the warehouse: they not only track the goods during moving them between stations, but also discover the remaining supplies and update their position, as well.

Nevertheless, future work is still ongoing in relation to similar live use-cases, which further highlight the advantages and eliminate the limitations of the presented solution.

As for wider perspectives, digital twins appear in all areas of production and logistics, hence the proceedings of digital-twin related research, development and innovation activities are expected to expand [7]. Associated with digital twins, mass individualization and *lot size one* paradigms are reshaping the production logics deep inside the manufacturing process level. Since smart assets are getting traceable on-the-fly, and their digital twin can contain not only status logs but actual production recipes for the asset, they can potentially drive autonomous production re-organization to meet the *lot size one* requirement on the spot. Such self-organization of manufacturing process for mass individualization [61] requires high flexibility and interoperability. This can be achieved by Service Oriented IoT Architectures, based on which the Arrowhead Framework also enables such workflow choreography [62].

For an even wider view, the "ABCDE5G" technologies—artificial intelligence, blockchains, cloud computing, big data analytics, edge computing, private 5G campus networks—are fostering the industrial IoT domain [63]. When it comes to asset traceability, blockchains and related technologies (such as distributed ledgers and smart contracts) also provide added value to data security traceability of smart assets. Some blockchain-based industrial models, such as ManuChain [64] and Makerchain [65] have already been proposed to address these very issues.

## Figures and Tables

**Table 1:** Schedule of Test Phases.

Time	Event
14:39:50	Start
14:40:20–14:40:30	Arrival at workstation A
14:41:10–14:41:20	Stopping at the obstacle
14:41:50–14:42:00	Arrival at workstation B
14:42:30	Leaving workstation B

**Table 2:** Summary of the Use-Case Scenario: IMU Triggers UWB Ranging, the RFID Reader Identifies the Assets in Sight (Numbers in Brackets Mark Measurements, That Ore Not Part of the Identification Scheme). Current State Stands for the Mode the System Operates in at the Moment, Asset Tracking Events Describe The Outcome of the Operation.

Time	IMU Trigger	UWB Ranging	Number of RFID Tags	Current State	Asset Tracking Events
14:39:50			(0)	STAND–BY	
14:40:00			(0)	STAND–BY	
14:40:10			(0)	STAND–BY	
14:40:20	X		(3)	START	
14:40:30		X	2	MOTION	new products detected
14:40:40			4	STOP	new products detected at A
14:40:50			(4)	STAND–BY	
14:41:00			(3)	STAND–BY	
14:41:10	X		(3)	START	
14:41:20		X	2	MOTION	products are taken
14:41:30		X	2	MOTION	products are taken
14:41:40			2	STOP	products arrived at obstacle
14:41:50	X		(2)	START	
14:42:00		X	4	MOTION	new products detected
14:42:10		X	4	MOTION	new products detected
14:42:20			4	STOP	products arrived at B
14:42:30	X		(4)	START	
14:42:40		X	0	MOTION	empty forklift
14:42:50			0	STOP	empty forklift
14:43:00			(0)	STAND–BY	
